# 

**Published:** 1867-06

**Authors:** 


### Dental Register.

EDITED BY

J. TAFT & G. WATT.

JUNE, 1867.

MONTHLY:
THREE DOLLARS PER ANNUM, IN ADVANCE.

CINCINNATI:
WRIGHTSON & CO., Printers, 167 WALNUT STREET.

*J. & C. R. TAFT, Dentists, 238 West Fourth St.*

